# *FMR1* CGG repeat expansion mutation detection and linked haplotype analysis for reliable and accurate preimplantation genetic diagnosis of fragile X syndrome

**DOI:** 10.1017/erm.2017.10

**Published:** 2017-07-19

**Authors:** Indhu-Shree Rajan-Babu, Mulias Lian, Felicia S.H. Cheah, Min Chen, Arnold S.C. Tan, Ethiraj B. Prasath, Seong Feei Loh, Samuel S. Chong

**Affiliations:** 1 Department of Pediatrics, Yong Loo Lin School of Medicine, National University of Singapore, Singapore 119074, Singapore; 2 Preimplantation Genetic Diagnosis Centre, Khoo Teck Puat – National University Children's Medical Institute, National University Health System, Singapore 119228, Singapore; 3 Thomson Fertility Centre, Thomson Medical Centre, Singapore 307470, Singapore; 4 Molecular Diagnosis Centre and Clinical Cytogenetic Laboratory Services, Department of Laboratory Medicine, National University Hospital, National University Health System, Singapore 119074, Singapore

## Abstract

*Fragile X mental retardation 1* (*FMR1*) full-mutation expansion causes fragile X syndrome. Trans-generational fragile X syndrome transmission can be avoided by preimplantation genetic diagnosis (PGD). We describe a robust PGD strategy that can be applied to virtually any couple at risk of transmitting fragile X syndrome. This novel strategy utilises whole-genome amplification, followed by triplet-primed polymerase chain reaction (TP-PCR) for robust detection of expanded *FMR1* alleles, in parallel with linked multi-marker haplotype analysis of 13 highly polymorphic microsatellite markers located within 1 Mb of the *FMR1* CGG repeat, and the *AMELX/Y* dimorphism for gender identification. The assay was optimised and validated on single lymphoblasts isolated from fragile X reference cell lines, and applied to a simulated PGD case and a clinical in vitro fertilisation (IVF)-PGD case. In the simulated PGD case, definitive diagnosis of the expected results was achieved for all ‘embryos’. In the clinical IVF-PGD case, delivery of a healthy baby girl was achieved after transfer of an expansion-negative blastocyst. *FMR1* TP-PCR reliably detects presence of expansion mutations and obviates reliance on informative normal alleles for determining expansion status in female embryos. Together with multi-marker haplotyping and gender determination, misdiagnosis and diagnostic ambiguity due to allele dropout is minimised, and couple-specific assay customisation can be avoided.

## Introduction

CGG-triplet repeat hyperexpansions in the 5′ untranslated region of the X-linked *fragile X mental retardation 1* (*FMR1*) gene cause fragile X syndrome (OMIM #300624) via *FMR1* promoter hypermethylation and sequential loss of the encoded FMR1 protein (Refs 1–3). Fragile X syndrome is the most common single-gene heritable form of intellectual disability, and the causative full-mutation (>200 CGGs) has an estimated prevalence rate of ~1 in 4000 males and 1 in 5000–8000 females (Refs 4, 5). Affected individuals usually inherit a copy of the mutant *FMR1* allele from their mothers, who harbour a full-mutation or an unstable premutation (55−200 CGGs) that is prone to undergo full-mutation expansion during intergenerational transmission. The risk of premutation-to-full-mutation allele transition is largely influenced by the maternal *FMR1* allele size, with ~100% full-mutation inheritance risk reported among offspring of women harbouring ≥100 CGGs (Ref. 6). In addition, ~21% of the premutation females experience fragile X-associated primary ovarian insufficiency (FXPOI) (Ref. 7), a condition that results in infertility and onset of menopause before the age of 40. At-risk couples can avoid fragile X syndrome-affected/carrier pregnancies by preimplantation genetic diagnosis (PGD) of in vitro fertilisation (IVF)-derived embryos and selective transfer of embryo(s) with normal (5−44 CGGs) *FMR1* allele(s) for implantation.

PGD for fragile X syndrome currently involves *FMR1* molecular analysis by polymerase chain reaction (PCR) amplification across the CGG repeat, either directly from a single blastomere or after whole-genome amplification (Refs 8–14). A major limitation of this repeat-spanning PCR approach relates to its inability to amplify large GC-rich premutation and full-mutation *FMR1* alleles, making it difficult to discern homozygous normal female embryos from those heterozygous for a normal allele and a nonamplifiable premutation or full-mutation allele. Consequently, repeat-spanning PCR analysis can only be offered to the estimated 64% of fragile X syndrome couples whose normal *FMR1* alleles are informative (i.e. the maternal and paternal normal alleles differ in CGG repeat size) (Ref. 14). In contrast, the triplet-primed PCR (TP-PCR) method (Ref. 15) enables consistent detection of *FMR1* expansions from both sexes and effectively resolves zygosity issues in females, and is thus applicable to all couples even if their normal alleles are uninformative. TP-PCR has been used in routine fragile X syndrome molecular diagnostic testing (Refs 16–20) and has been applied in the PGD of myotonic dystrophy type 1, another trinucleotide repeat hyperexpansion disorder (Refs 8, 15, 21), but has not been reported for fragile X syndrome PGD.

Due to the possibility of locus-specific amplification failure and/or allele dropout that is inherent in PCR-based assays or protocols involving whole-genome amplification (Ref. 22), concurrent haplotype analysis of flanking microsatellite or short tandem repeat (STR) markers has been employed to supplement direct mutation detection to minimise instances of misdiagnosis and inconclusive diagnosis. We have developed a fragile X syndrome PGD strategy that couples direct detection of the *FMR1* CGG repeat expansion by TP-PCR, with linked multi-marker haplotype analysis and gender determination using a tetradecaplex STR PCR assay that we recently described (Ref. 23). We have successfully applied this combined *FMR1* TP-PCR and tetradecaplex marker PCR assay to a simulated PGD case and two cycles of a clinical IVF-PGD case.

## Materials and methods

### Biological samples

Single cells isolated from lymphoblastoid cell lines (Coriell Cell Repositories, CCR; Camden, New Jersey, USA) were used to validate the *FMR1* TP-PCR and tetradecaplex marker PCR assays for direct CGG repeat and linked multi-marker haplotype analyses, respectively. Isolation, lysis and/or whole-genome amplification, using the Genomiphi^™^ V2 DNA (GE Healthcare, Little Chalfont, UK) or REPLI-g (Qiagen, Hilden, Germany) Amplification Kit, of single lymphoblasts were performed as described (Ref. 24). This study was approved by the Institutional Review Board of the National University of Singapore (B-15-273E).

### PGD cases

Archived samples of a simulated PGD case from the UK National External Quality Assessment Schemes (UK NEQAS) for Molecular Genetics (2011–2012), as well as embryos from two cycles of a clinical IVF-PGD case, were analysed using the validated assays. The simulated PGD case consisted of genomic DNA of a premutation carrier female (~31/~77 CGGs), her husband (~24 CGGs), an affected son (~480 CGGs), as well as two blastomeres from each of five embryos. Archived genomic DNAs of the parent-son trio and single-cell whole-genome amplification products of the blastomeres were analysed in parallel by *FMR1* TP-PCR and tetradecaplex marker PCR.

The clinical IVF-PGD case involved a premutation carrier female (34/60 CGGs), her husband (37 CGGs) and a premutation carrier daughter (37/62 CGGs). A total of 33 oocytes were recovered from two IVF-PGD cycles, of which 25 were mature and underwent intracytoplasmic sperm injection. Sixteen embryos fertilised successfully and were subjected to biopsy. Isolated embryo samples were subjected to whole-genome amplification and tested concurrently by *FMR1* TP-PCR and tetradecaplex marker PCR. Genomic DNAs of the parent-daughter trio were included to establish haplotype phase of the normal and mutant X chromosomes. Informed consent was obtained from the couple during initial consultation and IVF-PGD was performed under the Health Services Development Program, Ministry of Health, Singapore.

### PCR amplification

The tetradecaplex marker PCR was performed as described by Chen et al. (Ref. 23). *FMR1* TP-PCR and/or conventional repeat-spanning PCR was performed in a 50 µl reaction mix containing 5 U HotStarTaq DNA polymerase (Qiagen), 2.5× Q Solution (Qiagen), 1× supplied PCR buffer (Qiagen), 0.2 mm deoxyribonucleotide triphosphates (Roche, Penzberg, Germany) and either 10 or 100 ng genomic DNA, 6 µl single-cell lysate or 2 µl whole-genome amplification product as template. Conventional PCR across the CGG repeat utilised 0.4 µm each of primers, 5′-F1 (5′-TTCGGTTTCACTTCCGGTGGAGGGCCGCCT-3′) (Ref. 25) and *Fam*-*f* (5′-AGCCCCGCACTTCCACCACCAGCTCCTCCA-3′) (Ref. 26). The TP-PCR protocol was adapted from Teo et al. (Ref. 25) and utilised 0.4 µm each of primers *Fam*-*f* and Tail-F (5′-TACCGATACGCATCCCAGTTTGTCAGC-3′), and 0.04 µm of primer TP-F (TACCGATACGCATCCCAGTTTGTCAGC(CGG)_5_). Both repeat-spanning PCR and TP-PCR were performed on the GeneAmp^®^ PCR System 9700 (Applied Biosystems-Life Technologies, Carlsbad, CA). Thermal cycling included an initial denaturation at 95°C for 15 min, followed by 35 (for genomic DNA or single-cell whole-genome amplification product) or 45 (for single cells) cycles of 99°C for 2 min, 65°C for 2 min and 72°C for 3 min and a final extension at 72°C for 10 min.

For analysing repeat-spanning PCR amplicons, a 2-μl aliquot of *Fam*-labeled PCR product was mixed with 0.4 µl of MapMarker^®^ 1000 ROX (Bioventures, Murfreesboro, TN) internal size standard and 9 µl of Hi-Di^™^ Formamide (Applied Biosystems), denatured at 95°C for 5 min and subjected to CE (capillary electrophoresis) (36 cm, POP-7^™^, 18 s 1.2 kV injection, 50 min 15 kV run) on the 3130*xl* Genetic Analyser (Applied Biosystems). TP-PCR amplicons were also processed for CE analysis in a similar manner, with a modified electrophoretic run parameter (36 cm, POP-4^™^ or POP-7^™^, 18 s 1.2 kV injection, 30 min 15 kV run) and different internal size standard (0.4 µl of GeneScan^™^ 500 ROX^™^, Applied Biosystems). GeneScan electropherograms were analysed using GeneMapper^®^ software (Applied Biosystems, version 4.0).

### Data interpretation

**Figure 1.**
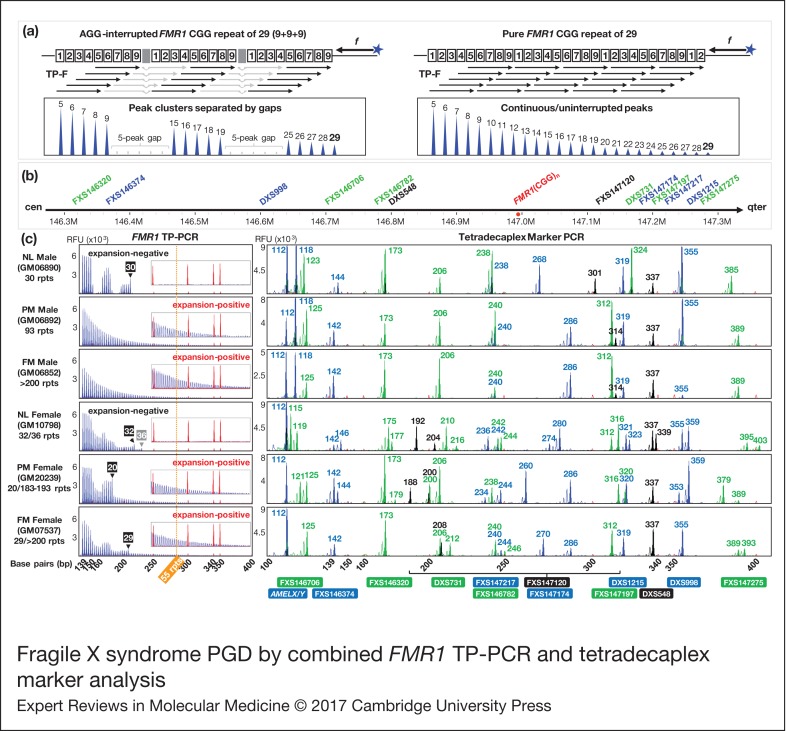
Fragile X syndrome PGD by combined *FMR1* TP-PCR and tetradecaplex marker analysis. (a) *FMR1* TP-PCR schematic depicting the annealing pattern of TP-F primer according to CGG repeat structure, and expected electropherograms. Shaded boxes indicate AGG interruptions. (b) Schematic showing STR marker loci relative to *FMR1* (CGG)_*n*_. (c) *FMR1* TP-PCR and tetradecaplex marker PCR electropherograms of representative whole-genome amplified single lymphoblasts. CGG repeat sizes of normal allele(s) determined by TP-PCR are indicated in numbered black/gray boxes. The 55-repeat cut-off for *FMR1* expansion detection is represented by a vertical dotted orange line. Numbers in the tetradecaplex marker PCR electropherograms indicate amplicon fragment sizes in bp, and STR names are indicated below their corresponding allele peaks. Red peaks in electropherograms are from the ROX-labeled internal size calibrator. *FMR1, Fragile X mental retardation 1*; TP-PCR, triplet-primed polymerase chain reaction; NL, normal; PM, premutation; FM, full-mutation; rpts, repeats, including AGG interruptions; RFU, relative fluorescence units.

*FMR1* TP-PCR generates a continuous series of amplicon peaks that differ from each other by three base pairs (bp), due to random annealing of the triplet-primed primer (TP-F) to the CGG repeat. As shown in Fig. 1a, a CGG repeat with AGG interruptions will generate discrete peak clusters with gaps of ~18 bp (equivalent to five missing peaks); the gaps being caused by the inability of primer TP-F to anneal over an AGG interruption. In contrast, an uninterrupted CGG repeat allows TP-F to anneal anywhere within the repeat and generate a continuous stretch of amplicon peaks without any gaps. The first amplicon peak is generated by TP-F annealing to the last five CGGs at the 3′end of the repeat and represents a 5-repeat amplicon, and each succeeding amplicon peak contains one additional repeat. Repeat sizes of alleles are derived by counting observed plus missing TP-PCR peaks.

The tetradecaplex marker panel comprises 13 STRs whose positions relative to *FMR1* (CGG)_*n*_ are shown in Fig. 1b, together with the *AMELX/Y* dimorphism. Females will display only the *AMELX* amplicon, while males will additionally display the *AMELY* amplicon, which is 6 bp larger than *AMELX*.

## Results

### Comparison of conventional repeat-spanning PCR and TP-PCR

**Figure 2.**
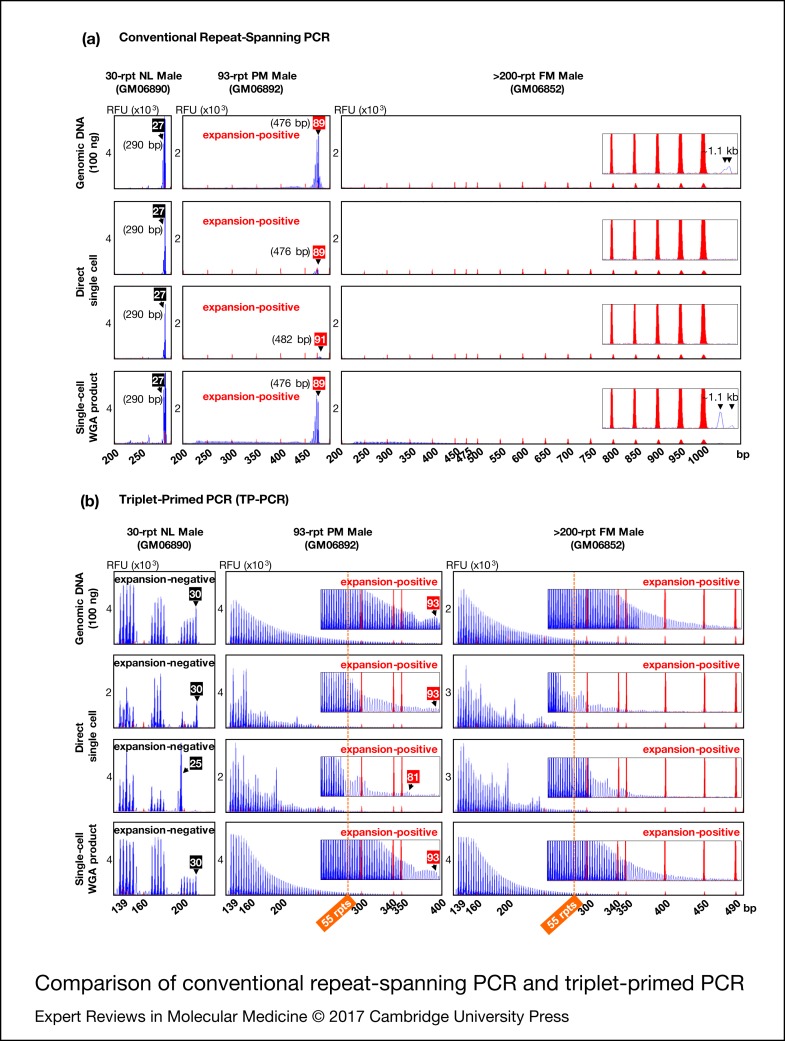
Comparison of conventional repeat-spanning PCR (a) and triplet-primed PCR (b). Black and red numbered boxes indicate the repeat sizes of normal and premutation alleles, respectively. TP-PCR, triplet-primed polymerase chain reaction. NL, normal; PM, premutation; FM, full-mutation; WGA, whole-genome amplification, rpt/rpts, repeats, including AGG interruptions.

To compare the utility of conventional repeat-spanning PCR and TP-PCR for fragile X syndrome PGD we performed the assays directly on single cells as well as on single-cell whole-genome amplification product from normal, premutation and full-mutation male cell lines. For reference, the assays were also performed on 100 ng genomic DNA (Fig. 2).

Repeat-spanning PCR of genomic DNA, single cells and single-cell whole-genome amplification product from the 30-repeat normal male cell line (GM06890) each produced a 290 bp amplicon peak (Fig. 2a). For the 93-repeat premutation male cell line (GM06892), repeat-spanning PCR produced a 476 bp amplicon peak in all samples except for one single cell which displayed a 482 bp peak, likely reflecting some mitotic instability of this premutation cell line. In addition, the premutation amplicon peak from single cells was of significantly lower fluorescence intensity compared to the amplicon peaks from genomic DNA and single-cell whole-genome amplification product.

When the repeat-spanning PCR amplicon sizes were converted into CGG repeats using the formula [*X*−209]/3, where *X* is the amplicon size in bp and 209 is the target region length excluding the CGG repeat sequence, the lengths of the 30-repeat normal and 93-repeat premutation alleles were underestimated as 27 and 89/91 CGGs, respectively. This discrepancy between actual and repeat-spanning PCR computed CGG repeat sizes can be ascribed to the electrophoretic migration anomalies of GC-rich *FMR1* amplicon fragments. Thus, unless an allelic size ladder is generated using sequence-verified controls to correct for this mobility shift, repeat-spanning PCR-based PGD assays may underestimate CGG repeat size and incorrectly genotype embryos carrying a borderline *FMR1* allele in the low-intermediate (or gray zone, 45−54 CGGs) and low-premutation repeat size ranges.

Repeat-spanning PCR on single cells of the full-mutation male cell line (GM06852) failed to detect the full-mutation expansion (>200 CGGs). When repeat-spanning PCR was performed on genomic DNA or single-cell whole-genome amplification product, however, a barely discernible broad peak was observed migrating at ~1.1 kb, possibly generated from heterogeneous extension products of the full-mutation allele.

In comparison with repeat-spanning PCR, the TP-PCR method determines CGG repeat sizes by simply counting the number of electrophoretic peaks generated, and provides information on the number and position of AGG interruptions within normal to small premutation alleles (Fig. 2b). Normal and premutation alleles of GM06890 and GM06892, respectively, were accurately sized from genomic DNA and single-cell whole-genome amplification product, while the full-mutation allele of GM06852 was visualised as a continuous stretch of amplicon peaks extending well beyond 55 repeats (the smallest expanded allele size), with peak fluorescence intensities tapering gradually to background fluorescence at ~400–490 bp (~95–125 CGGs). Direct TP-PCR of single cells also generated amplicon peaks and facilitated the differentiation between normal and expanded premutation and full-mutation alleles. However, the irregular peak patterns complicated repeat size and/or structure determination.

Overall, TP-PCR performed best at detecting *FMR1* CGG repeat expansions, especially when single-cell whole-genome amplification product was used as template.

### Validation of *FMR1* TP-PCR and tetradecaplex marker PCR assays on single lymphoblasts

To validate the combination of *FMR1* TP-PCR and tetradecaplex marker PCR (Ref. 23) for fragile X syndrome PGD, single lymphoblasts from normal, premutation and full-mutation male and female cell lines were subjected to whole-genome amplification and aliquots were used to perform the two assays in parallel. *FMR1* TP-PCR produced consistent amplification and detection of the expected normal, premutation and full-mutation alleles, while all 13 markers and *AMELX/Y* amplified successfully (Fig. 1c). The *FMR1* CGG repeat expansions present in GM06892, GM06852, GM20239 and GM07537 were successfully detected, as indicated by the series of amplicon peaks extending well beyond the 55-repeat premutation cut-off. *FMR1* TP-PCR also enabled precise determination of the number and position of AGG interruptions within the normal allele(s) of GM06890, GM10798, GM20239 and GM07537. Furthermore, the results from single-lymphoblast whole-genome amplification product were consistent with those obtained using cell line-derived genomic DNA (Ref. 19), thus indicating that *FMR1* TP-PCR can be used with confidence on single-cell whole-genome amplification product. GM10798, an α-thalassemia reference sample, was selected as the female sample with normal *FMR1* alleles, as it is heterozygous for all 13 linked STRs.

### Simulated PGD

**Figure 3.**
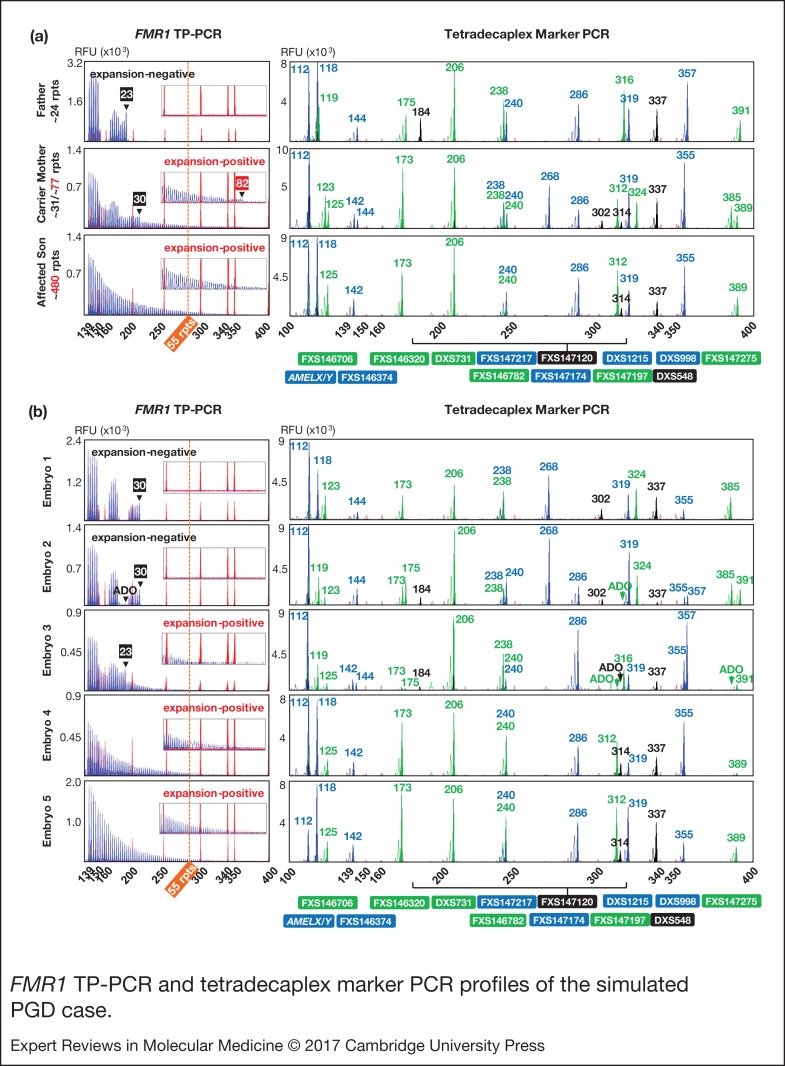
*FMR1* TP-PCR and tetradecaplex marker PCR profiles of the simulated PGD case. (a) *FMR1* TP-PCR and tetradecaplex marker PCR profiles of parent-son trio genomic DNA samples. (b) *FMR1* TP-PCR and tetradecaplex marker PCR profiles of whole-genome amplified ‘blastomeres’. Electropherograms of one of the two genotyped blastomeres of each ‘embryo’ are shown. ADO, allele dropout; *FMR1, Fragile X mental retardation 1*; TP-PCR, triplet-primed polymerase chain reaction.

We next applied the *FMR1* TP-PCR and tetradecaplex marker PCR to archived genomic DNA samples from a previous UK NEQAS proficiency test, consisting of a parent-offspring trio (unaffected father, carrier mother and affected son) (Fig. 3a) and archived whole-genome amplification product from simulated embryo blastomeres (Fig. 3b). TP-PCR confirmed the presence of an expanded allele in mother and son (Fig. 3a, left). The normal allele sizes of the parent-son trio were found to be one repeat smaller than what was indicated by UK NEQAS, whereas the premutation allele of carrier mother was determined to be five repeats larger than the indicated ~77 CGGs, probably due to differences in the repeat sizing methods employed.

The tetradecaplex marker PCR successfully amplified all 13 STRs and *AMELX/Y* from the DNAs of the parent-son trio. Four STRs (FXS146706, FXS147120, FXS147197 and FXS147275) were fully informative (all three parental alleles are dissimilar) and another four (FXS146374, FXS146782, FXS147174 and FXS147217) were semi-informative (one of the two maternal alleles is identical to the paternal allele) (Fig. 3a, right).

Of the five simulated embryos, the *FMR1* TP-PCR profiles of embryos 3, 4 and 5 displayed amplicon peaks that extend beyond the 55-repeat cut-off, indicating that they are ‘expansion-positive’ (Fig. 3b, left). An expanded allele was not detected in embryos 1 and 2.

The tetradecaplex marker PCR profiles of the five simulated embryos are presented in Fig. 3b (right). The presence of only a single allele for all 13 STRs, and the presence of both the *AMELX* and *AMELY* fragments, indicate that embryos 1, 4 and 5 are male, while the presence of two alleles for some STRs, and the presence of only *AMELX* indicate that embryos 2 and 3 are female. This being so, the TP-PCR result of embryo 2 should have detected a 23-repeat paternal normal allele, but this was not observed, most likely due to allele dropout. However, the presence of the 30-repeat maternal normal allele was sufficient to confirm this embryo's expansion-negative status.

**Figure 4.**
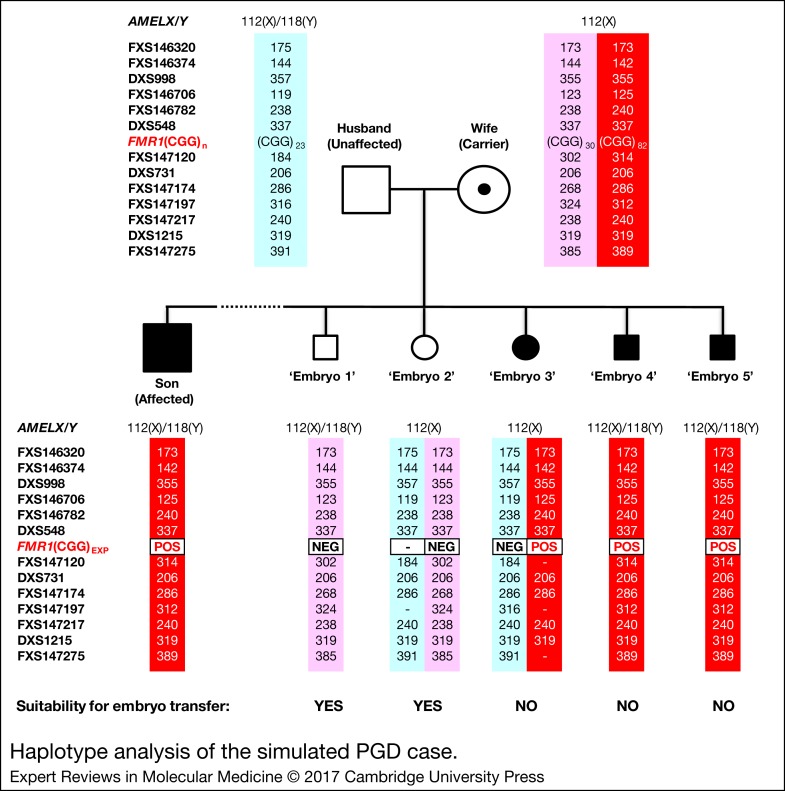
Haplotype analysis of the simulated PGD case. Haplotypes of father (unaffected), mother (carrier), son (fragile X syndrome-affected) and five ‘embryos’ are shown. Light- and dark-shaded numbered columns denote marker haplotypes linked to normal and mutant *FMR1* alleles, respectively. Haplotyped markers are ordered from centromere (top) to telomere (bottom) of q-arm. *FMR1* CGG repeat expansion status of each embryo is denoted as POS (positive) or NEG (negative). Dashes (–) indicate allele dropout, and numbers indicate STR amplicon size in bp. The haplotype linked to the maternal *FMR1* mutant allele was present in embryos 3, 4 and 5, while embryos 1 and 2 inherited the maternal *FMR1* normal allele. *FMR1, Fragile X mental retardation 1*.

The marker haplotype linked to the maternal premutation allele was determined directly from the hemizygous haplotype of the index fragile X syndrome-affected son (Fig. 4). As expected, the maternal mutant haplotype was observed in the three expansion-positive embryos (3, 4 and 5), but not in the expansion-negative embryos (1 and 2). Some allele dropout was observed, however, the high number of panel markers ensured that the occasional marker allele dropout or amplification failure did not adversely affect haplotype assignment.

Overall, the *FMR1* TP-PCR genotypes were consistent with the tetradecaplex marker PCR haplotypes, and the diagnoses were in complete agreement with the UK NEQAS report on these samples.

### Clinical IVF-PGD

**Figure 5.**
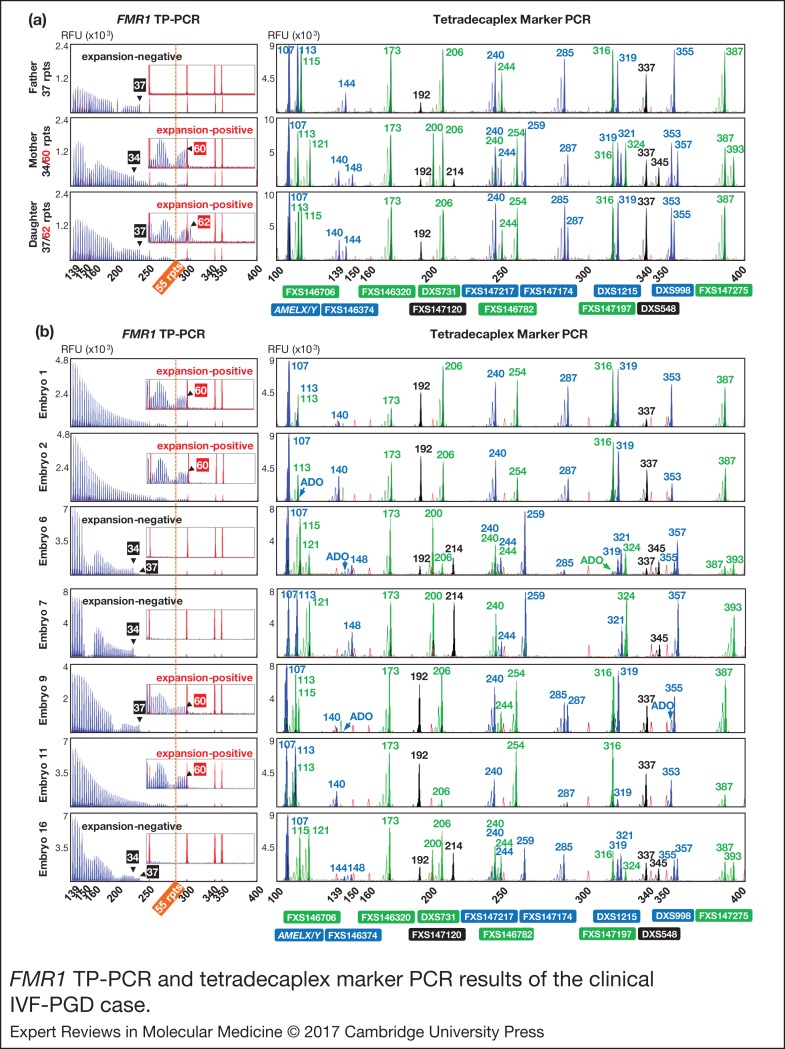
*FMR1* TP-PCR and tetradecaplex marker PCR results of the clinical IVF-PGD case. (a) *FMR1* TP-PCR and tetradecaplex marker PCR profiles of parent-daughter trio genomic DNA samples. (b) Representative *FMR1* TP-PCR and tetradecaplex marker PCR profiles of seven whole-genome amplified embryo blastomeres. Except for embryo 16, electropherograms of one of the two genotyped blastomeres are shown. *FMR1, Fragile X mental retardation 1*; TP-PCR, triplet-primed polymerase chain reaction.

The validated *FMR1* mutation detection and linked haplotype assays were clinically applied to a couple who had requested for fragile X syndrome PGD. The CGG repeat sizes and AGG interruption patterns, as well as STR marker genotypes, were determined in the parent-daughter trio. The couple was informative for their normal *FMR1* alleles, and the 60-repeat maternal premutation allele was observed to have expanded by two CGGs in the daughter (Fig. 5a, left). The tetradecaplex marker PCR showed successful amplification of all 13 STRs and *AMELX/Y* (Fig. 5a, right). The couple was fully informative for five markers (FXS146374, DXS998, FXS146706, FXS146782 and FXS147174), and semi-informative for another seven (DXS548, FXS147120, DXS731, FXS147197, FXS147217, DXS1215 and FXS147275). Only FXS146320 was uninformative. Linked marker haplotypes were constructed by comparing the individual marker genotypes of the carrier mother, normal father and carrier daughter.

The couple underwent two IVF-PGD cycles. In cycle 1, six of eight embryos developed sufficiently by day 3 for biopsy and analysis. The two slower growing embryos eventually developed into a morula and a blastocyst, and were vitrified. Four embryos (1, 2, 4 and 5) carried the maternal premutation allele and were excluded from transfer consideration. Embryos 3 and 6 (expansion-negative females) were transferred, but did not result in pregnancy.

In cycle 2, all eight embryos developed sufficiently by day 3 for biopsy and analysis. In addition, the two frozen embryos from cycle 1 were thawed, biopsied and analysed. Four fresh embryos (9, 11, 13 and 14) were positive for the maternal premutation allele and were excluded from transfer consideration. Also excluded from transfer consideration were fresh embryos 8 and 10, which experienced maternal allele dropout at 11 and eight STRs, respectively, and the maternal *FMR1* normal allele, as well as frozen-thawed embryo 15, which experienced paternal allele dropout at nine STRs and the paternal *FMR1* allele. Fresh embryos 7 and 12, and the frozen-thawed blastocyst (embryo 16) were definitively diagnosed as expansion-negative. Embryo 12 was subsequently arrested, while embryo 7 was grown to blastocyst and vitrified for future transfer. Frozen-thawed embryo 16, which had been re-frozen after biopsy in cycle 2, was re-thawed and transferred in a subsequent natural cycle, which resulted in the birth of a healthy baby girl. No molecular analysis has been performed on the newborn as the mother declined confirmatory testing.

**Figure 6.**
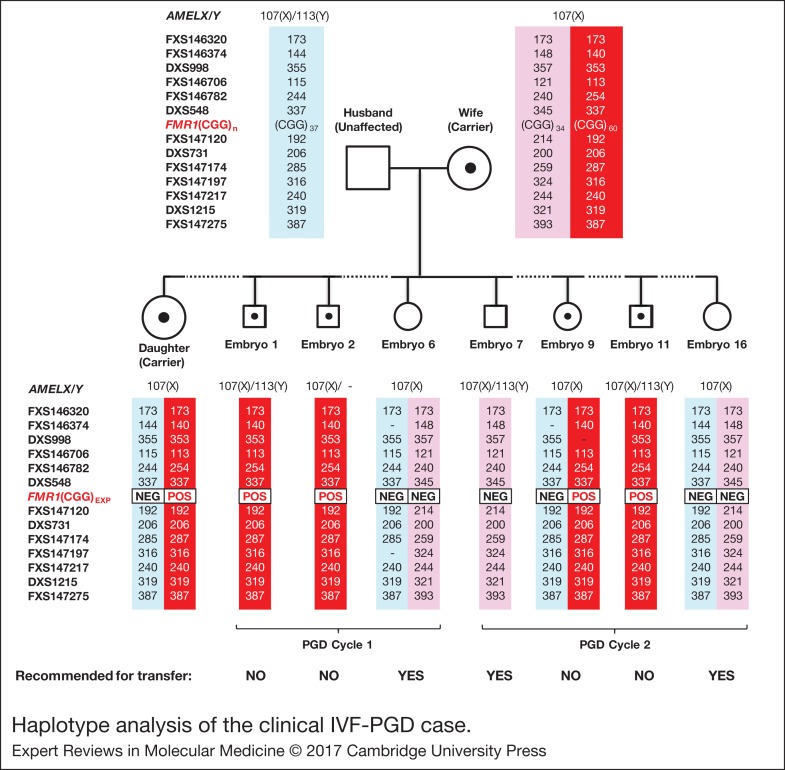
Haplotype analysis of the clinical IVF-PGD case. Haplotypes of father (unaffected), mother (carrier), daughter (carrier) and seven embryos from cycles 1 and 2 are shown.

The electropherograms of representative expansion-positive and expansion-negative male and female embryos are shown in Fig. 5b. There was good concordance between the *FMR1* TP-PCR and tetradecaplex marker PCR results, whereby embryos positive for the maternal CGG repeat expansion by TP-PCR also displayed the haplotype linked to the *FMR1* mutation (Fig. 6).

## Discussion

Fragile X syndrome PGD usually involves conventional PCR across the *FMR1* CGG repeat. As hyperexpanded alleles are refractory to repeat-spanning PCR amplification, especially when performed directly on single cells, this method was primarily aimed at detecting the nonexpanded parental allele(s) in normal embryos. However, when the paternal and maternal normal alleles are identical in repeat size, this method cannot distinguish homozygous normal from fragile X syndrome-affected female embryos, consequently restricting its application only to couples with informative normal *FMR1* alleles (Ref. 27). In marked contrast, TP-PCR (Ref. 15) resolves zygosity issues in females by generating a characteristic extended TP-PCR amplicon peak pattern in the presence of an *FMR1* expansion, regardless of repeat size. Although routinely used in diagnostic testing for fragile X syndrome (Refs 16–20), *FMR1* TP-PCR for single-cell fragile X syndrome PGD has not been reported.

We have optimised a *FMR1* TP-PCR for direct CGG repeat expansion detection that can be applied to direct analysis of single cells and single-cell whole-genome amplification products. As expected, the single lymphoblasts from premutation and full-mutation cell lines generated a characteristic extended TP-PCR amplicon peak pattern. TP-PCR allowed for more reliable CGG repeat size evaluation while dispensing with the need for allelic size ladders and mobility shift correction associated with repeat-spanning PCR due to the tendency of GC-rich amplicons to form higher-order structures that may increase their mobility relative to DNA size standards (Refs 28–31).

Allele dropout, in which one of two alleles at a heterozygous locus randomly fails to amplify, is commonly observed in direct or whole-genome amplification-based PCR amplification. Whole-genome amplification procedures using multiple displacement amplification have an average allele dropout rate, *a* of 25% (Ref. 27). At this rate, the probability that both *FMR1* alleles present in a heterozygous blastomere will be amplified is ~56% [(1 − *a*)^2^] (Refs 27, 32). Dropout of the maternal *FMR1* allele can result in inconclusive diagnosis and consequently reduce the percentage of embryos with a definitive diagnosis and ultimately the pregnancy success rate.

A high incidence of inconclusive results is particularly undesirable for FXPOI-affected premutation carrier women, who have poorer oocyte retrieval rates and comparatively fewer embryos available for PGD compared with their unaffected counterparts of the same age (Ref. 14). Furthermore, CGG repeat analysis alone is incapable of detecting the common sex chromosome aneuploidies Klinefelter syndrome (47, XXY; 1/500–1/1000 males) and Turner syndrome (45, X; 1/2500 females) when the paternal and maternal *FMR1* normal alleles are identical. Mosaic Turner syndrome is also commonly observed among fragile X syndrome-affected females, due to a predisposition for loss of the full-mutation-carrying chromosome during mitosis (Ref. 33). By coupling the direct mutation test with an indirect linked marker haplotype analysis, these deficiencies can be addressed. The reliability of linkage analysis, however, relies on the number of markers tested, their proximity to the *FMR1* CGG repeat and level of informativeness, and to some extent on the availability of a related index case for haplotype phasing. Even when an index case is unavailable to infer the mutant haplotype, in combination with the *FMR1* TP-PCR, it is possible to ascertain the maternal normal and mutant haplotypes when an expansion-negative or expansion-positive male embryo is present.

We recently developed a single-tube multi-marker PCR that co-amplifies 13 highly polymorphic STRs located <1 Mb from the *FMR1* CGG repeat, along with *AMELX/Y* fragment for gender determination (Ref. 23). Besides minimising chances of indeterminate diagnosis resulting from marker-mutation recombination to <1%, the multiple highly polymorphic STRs in the panel significantly reduce the likelihood of misdiagnosis and inconclusive diagnosis due to allele dropout or exogenous DNA contamination, and virtually dispenses with the need for couple-specific customisation of informative markers. Furthermore, the flexibility of the tetradecaplex marker assay to be performed directly on a single cell or on single-cell whole-genome amplification product makes it among the most versatile multi-marker fragile X syndrome PGD assays reported thus far (Ref. 23).

By combining the single-cell *FMR1* TP-PCR for direct detection of CGG repeat expansion mutation with the tetradecaplex marker PCR for linkage-based analysis of flanking polymorphic markers, a higher diagnostic confidence with fewer inconclusive outcomes is achieved. Two PGD cases (a simulated PGD case from UK NEQAS and a clinical IVF-PGD case) were analysed using this combined assay. In both cases, the *FMR1* TP-PCR and tetradecaplex marker PCR complemented each other in detecting embryos that were positive for the maternal mutant *FMR1* allele. For both simulated and clinical PGD cases, four to five STRs were fully informative, indicating that the tetradecaplex STR panel can yield a diagnostic power of 96.3–98.4% (1 −  [1 − (1 − *a*)^2^]^*N*^, *N* being the number of fully informative markers) (Ref. 32), assuming an *a* of 25%. No additional case-specific customisation of the marker panel was required. The additional data from the semi-informative markers together with the analysis of two cells per embryo contributed to achieving a conclusive diagnosis in all tested embryos, except for three embryos from the clinical IVF-PGD as mentioned above.

It should be noted that the *FMR1* TP-PCR does not distinguish large premutation (95−200 CGGs) from full-mutation (>200 CGGs) alleles. Maternal premutation allele size and number of AGG interruptions may predict likelihood of expansion to full-mutation upon transmission to the embryo (Refs 6, 34). For instance, when the mother carries a large premutation allele (>90 CGGs) with no AGG interruptions, the likelihood that an ‘expansion-positive’ embryo has inherited a full-mutation allele is very high. Small expansions (<95 CGGs) can be precisely sized using the single-cell *FMR1* TP-PCR, and repeat instability during intergenerational transmission can be reliably assessed. In the clinical IVF-PGD case, the 60 CGG (9 + 9 + 40) maternal premutation allele was observed to have expanded by two CGGs in the carrier daughter. This observation is consistent with the Nolin et al. reports (Refs 34, 35), which state that ~50% of the 60−64-repeat maternal alleles with two AGG interruptions exhibit unstable transmissions with a median repeat change of approximately one to two CGGs. Though the risk of FM expansion in this clinical IVF-PGD case appears to be negligible, minor instability and limited expansion of the maternal premutation allele, and/or de novo loss of the repeat stabilising AGG interruption(s) in subsequent generations cannot be completely ruled out. PGD in this couple has eliminated trans-generational transmission of a potential fragile X syndrome-causing mutation, sparing future generations from worries over further expansion into the disease-causing range. The European Society for Human Reproduction and Embryology Task Force on Ethics and Law highlights that PGD for such nonmedical reasons may be morally justified and legitimate (Ref. 36).

This combined fragile X syndrome PGD assay increases diagnostic confidence through concordance analysis, minimises misdiagnosis caused by unobserved allele dropout, exogenous DNA contamination, or genetic recombination, minimises discard of potentially healthy embryos caused by inconclusive results and secondarily detects common sex chromosome aneuploidies. Given the high premutation prevalence in the general population (1/148 women) (Ref. 37) and the anticipated increase in requests for fragile X syndrome PGD as carrier screening programs become more widespread (Refs 38, 39), a robust PGD strategy that can be applied without modification to most, if not all, at-risk couples would be useful.
